# Collecting Knowledge for the Family: Recipes, Gender and Practical Knowledge in the Early Modern English Household

**DOI:** 10.1111/1600-0498.12019

**Published:** 2013-04-30

**Authors:** Elaine Leong

**Affiliations:** * Department II (Daston), Max Planck Institute for the History of ScienceBoltzmannstrasse 22, Berlin, 14195, Germanyeleong@mpiwg-berlin.mpg.de

**Keywords:** Early modern medicine, gender history, household, informal science

## Abstract

When Mary Cholmeley married Henry Fairfax in 1627, she carried to her new home in Yorkshire a leather-bound notebook filled with medical recipes. Over the next few decades, Mary and Henry, their children and various members of the Fairfax and Cholmeley families continually entered new medical and culinary information into this ‘treasury for health.’ Consequently, as it stands now, the manuscript can be read both as a repository of household medical knowledge and as a family archive. Focusing on two Fairfax ‘family books,’ this essay traces on the process through which early modern recipe books were created. In particular, it explores the role of the family collective in compiling books of knowledge. In contrast to past studies where household recipe books have largely been described as the products of exclusively female endeavors, I argue that the majority of early modern recipe collections were created by family collectives and that the members of these collectives worked in collaboration across spatial, geographical and temporal boundaries. This new reading of recipe books as testaments of the interests and needs of particular families encourages renewed examination of the role played by gender in the transmission and production of knowledge in early modern households.

## 1. Introduction

In the early 1880s, George Weddell, chemist and entrepreneur, moved his firm into new premises on 135 Pilgrim Street in Newcastle-upon-Tyne.^1^ During the general reorganization of the offices, Weddell discovered a small book of early modern medical and culinary recipes in a box of lumber.^2^ Intrigued on both a personal and professional level, Weddell embarked on a long journey to uncover the story of the notebook. Seven years later, Weddell's painstaking research led him to connect the book with the Yorkshire-based Fairfax family and, in particular, with Henry (1588–1667) and Mary Cholmeley Fairfax (1592–1649). Driven by his conviction that the manuscript was of interest to bibliophiles and antiquarians alike, Weddell privately published a hand-traced facsimile edition of the book as *Arcana Fairfaxiana Manuscripta*. With the original manuscript still to be located, it is this facsimile edition which makes Henry and Mary Fairfax's recipes and household knowledge available to modern readers.

Almost twenty-five years after Weddell's issue of the *Arcana Fairfaxiana*, a recipe book linked with Henry Fairfax's elder brother Ferdinando (1584–1648), parliamentarian general and politician and Ferdinando's second wife Rhoda found its way into the collection of another chemist entrepreneur – Sir Henry Wellcome.^3^ By this date, Wellcome's collecting enterprise was in full swing. Often acting under pseudonyms, Wellcome and his army of staff were attending several sales and negotiating numerous acquisitions every week (Larson, 2009, ch. 6). On 26 July 1907, Charles Thompson (1862–1943), medical historian, museum curator and agent to Wellcome, picked up a folio-sized leather-bound household recipe book from Sotheby's in Covent Garden, London. The notebook's connection to the Fairfax family is asserted through Rhoda Hussey Fairfax's two ownership notes on the front fly-leaf. In addition, while the metal clasps which once held the notebook closed are now missing, the volume retains its original bindings and the gild-stamped ornament with Rhoda's initials decorating the front and back covers (Fairfax Family).^4^

The two Fairfax family books are filled to the brim with practical knowledge useful for the running of early modern households. Flipping through, readers can find instructions for medicinal remedies, the pickling of vegetables and fruit, simple dishes such as gruels and pies, elaborate sugar crafts to impress guests, or household tips for cleaning linens and perfuming bottles. It was this miscellaneous quality of the Fairfax recipe books that captured the imagination of the two turn-of-the century chemists. Weddell saw Henry and Mary Fairfax's book as an opportunity to study and investigate everyday life in pre-modern households.^5^ Wellcome, on the other hand, was interested in these books as ‘curious medical lore.’ The collecting and safeguarding of family recipe books such as Rhoda Fairfax's grew out of his desire to write a definitive book on animal substances used in medicines and his grand scheme to create an ‘Historical Exhibition of Rare and Curious Objects relating to Medicine, Chemistry, Pharmacy and the Allied Sciences.’^6^

Recent studies by historians of science have continued to expand the parameters delineating the ‘spaces’ of early modern natural inquiry. Looking beyond traditional settings of universities and academies, places such as princely courts, markets, artisanal workshops and printing houses are now widely accepted as spaces in which knowledge was created.^7^ Within this literature, the early modern domestic space has come under increasing scrutiny as a site of knowledge production.^8^ Historians of medicine see the early modern home as one of the main sites for medical intervention and health promotion. Householders were not only quick to combine self-diagnosis and self-treatment with commercially available medical care but many also produced their own homemade medicines.^9^ Books such as the Fairfax recipe books were commonplace in early modern England and, alongside the rich offerings in vernacular medical print and household guides, provided readers with a framework of health-related knowledge to guide their home-based practices. For historians, these texts are some of the most revealing sources about home-based practices of natural inquiry. Though they concentrate on the practicalities of doing and making rather than presenting theoretical frameworks, they give a sense not only of householders' areas of interest in natural knowledge but also of the types of activities carried out within early modern homes.

Recipes, both medical and culinary, were the main medium for the recording and transmission of information and knowledge in pre-modern households. Many families had a designated notebook in which to record, create and communicate these necessary bits of practical knowledge and hundreds of such books now survive in archives and libraries, each representing the varying needs, interests and aspirations of generations of men and women. The gathering together of medical information in the form of short, concise recipes has a long tradition stretching from the ancient period to the late-nineteenth century when Victorian English families still brought their own customized recipes to be ‘made up’ at local pharmacies.^10^ The recent flurry of research on early modern recipe books and particularly those created in England, has resulted in a rich interdisciplinary historiography. Within this body of literature, recipe books, whether in manuscript or in print, have featured most prominently in accounts of domestic management, studies of women's medical practices and discussions of women's writings.^11^ Modern editions of recipe books have titles such as ‘Lady Sedley's Receipt Book,’ *Elinor Fettiplace's Receipt Book* and *Ladie Borlase's Receiptes Booke*, conjuring up a homey picture of the lady of the manor collecting all sorts of household information as part of her housewifely duties.^12^ The modern editors of these three seventeenth-century recipe books no doubt based their titles on ownership notes inscribed in the manuscripts. Yet these titles obscure the rich and complex stories of authorship and ownership connected with the texts. For example, while David E. Schoonover titled the modern edition of the Borlase family manuscript as *Ladie Borlase's Receiptes Booke*, he also acknowledged that the volume bears dates from 1665 to 1822 suggesting that it was not the work of just one Lady Borlase (Schoonover, 1998, p. 1). Schoonover's titling of his edition exemplifies the general view that many surviving early modern English manuscript household books are firstly, single-authored and secondly, authored by women. More nuanced readings have been offered by folklorist Janet Theophano and cultural historian Sara Pennell who acknowledged that cookbooks are communal affairs and that ‘culinary knowledge is collectively generated.’^13^ However, the main aim of both these studies was to analyze cookbooks as reflections of ‘female knowledge formation (Pennell, 2004, p. 242).’ Another scholar, Monica Green, in her sophisticated analysis of the ‘gendering of women's medicine,’ posits that the recipe book can be considered the first genre of women's medical writing (Green, 2008, p. 301). For Green, these books presented ‘a major shift in the role that literacy played within domestic medical traditions since the male dominance of the household book we witnessed earlier now gave way to a new feminine model (Green, 2008, p. 308).’

While recipe books undoubtedly present us with an opportunity to uncover women's involvement in household medicine and science, the histories and life stories of the Fairfax notebooks and similar texts challenge these female-oriented readings. Both the *Arcana Fairfaxiana* and Wellcome Western Manuscript 160 might, at first glance, appear to be associated with individual women, but once we scratch beyond the surface, we discover generations of men and women connected to and participating in the writing of their ‘family book.’ This essay, focused on untangling the processes through which household recipe books were created, highlights two distinctive features of the genre: multiple ownership and collaborative authorship. In doing so, it builds on recent studies by literary and textual historians on co- and social authorship.^14^ In particular it answers Helen Smith's call for us to adopt ‘an understanding of book creation as collaborative and contingent and…that all texts, not simply those attributed to women, were marked and mediated by numerous agents, rendering books more mobile and more complexly sexed than has been allowed (Smith, 2012, p. 6).’ Running throughout the essay is the contention that while early modern women were actively involved and played a significant role in the making of household knowledge, they were joined in these endeavors by their fathers, husbands, brothers and sons. By suggesting that communities of knowledge-collectors rather than single authors were behind the making of these books, I hope to further expand our purview of the actors conducting natural inquiry in early modern kitchens, stillrooms and closets.

## 2. Keeping It in the Family

While two nineteenth-century chemists, George Weddell and Henry Wellcome, might have taken the final steps to ensure the survival of the Fairfax recipe books into the present day, the pre-modern *fortuna* of both books was largely determined by marriage alliances and familial networks. Family was central to the creation and transmission of household practical knowledge. Inheritance and bequest within lineage family structures were central to the transmission of household recipe books, whereas the expansive household-family and kinship family relationships delineated the far-reaching stretches of a compiler's knowledge network.^15^ Aside from the Fairfax connections and their chance rescue by the collecting interests of two turn-of-the century pharmacist/entrepreneurs, the two recipe books at the center of this essay share a number of similar stages in their life cycles.

In the first stage, both books entered the lives of the Fairfax family through marriage. The *Arcana Fairfaxiana* accompanied Mary Cholmeley to her new home when she married Henry Fairfax in February 1627 (Hopper, 2004b). The manuscript's life before entering the Fairfax family is revealed by the initials ‘M. C.’ on the front and back covers linking the book to Mary or her mother Margaret Cholmeley. Wellcome Western 160 came to Yorkshire through Rhoda Chapman Hussey's second marriage to Ferdinando Fairfax in 1646. Like her sister-in-law Mary's recipe book, the book Rhoda brought with her had also begun life before her marriage into the Fairfax clan. The use of the initials ‘R. H.’ on the decorative front cover and the signature ‘Rhoda Hussey’ on the front fly-leaf suggests that Rhoda first obtained the book whilst married to her first husband Thomas Hussey of Lincolnshire.

Once the notebooks arrived in their new homes, they took on their role as repositories of the household's practical knowledge and were added to and extended during and beyond the lifetimes of Mary and Rhoda Fairfax. Throughout her life, Mary was aided in this endeavor by her partner in life and collaborator in recipe collecting, Henry Fairfax, who signals his role in the creation of the volume by his ownership note on the first folio. The key place Rhoda's recipe book played in her life after her marriage to Ferdinando Fairfax is demonstrated by her second signature on the fly-leaf as ‘Rhoda Fairfax’ and by a number of dated recipes, such as one for the ‘vatican pill’ described as ‘a pill recommended for mee R. F. by Doctor Catlin, 1653 when I was very ill’ and another, on a loose leaf slipped in later in the book with the addendum, ‘this was sent from London for the payne in my shoulder…R. F. the 29^th^ July 1682.’^16^

Finally, after the deaths of Mary, Henry and Rhoda, these two treasuries of practical knowledge continued to benefit at least another four generations of Fairfax men and women. In the *Arcana Fairfaxiana*, two other ownership notes, ‘Ursula Lister’ and ‘Robert Green of Cocken ex dono R. Carr,’ join that of Henry Fairfax (Fairfax Family, 1890, p. 1).^17^ Both Lister and Carr had connections to the Fairfax family. Ursula Lister was the daughter of Henry's cousin Sir William Fairfax of Steeton (Fairfax Family, 1890, p. xxix). Carr, on the other hand, is believed to be either the husband or the son of Henry's granddaughter Anne, who married Ralph Carr of Cocken and, in 1694, had a son who was also named Ralph (Fairfax Family, 1890, pp. xvi–ii). It was through one of these two men that the manuscript left the possession of the Fairfax family. How the book got from the home of Robert Green to the Newcastle offices of Gilpin and Company is unknown but what is certain is that the book was the prized possession of the Fairfax family for over a century.

Bequest and inheritance also played a pivotal role in determining the life story of Wellcome Western MS 160. Rhoda and Ferdinando's daughter, Ursula, inherited the book some time before 1665 and marked her ownership in the time-honored way of signing her name on the front fly-leaf. The book then accompanied Ursula to Aynho, Northamtonshire on her marriage to Thomas Cartwright. It is perhaps there that Ursula's stepdaughter, Dorothy Cartwright, also signed her name in the book (Longden, 1935, p. 42). After remaining with the Cartwrights at Aynhoe Park for two generations, Rhoda's book accompanied her great-granddaughter Ursula to Newbold Hall when she married Sir Francis Skipwith. The final journey taken by the volume from Newbold Hall to the Wellcome Collection was occasioned by Sir Grey Skipwith's desire (or probably need) to auction off his library in the early twentieth century. Evidently, the notebook once treasured by previous generations of the Fairfax and Cartwright families did not hold the same value for Sir Grey. Our ability to trace the *fortuna* of both manuscripts through the Fairfax family tree highlights how close familial relationships largely determine who was included in the privileged circle permitted to share and augment the family treasury of practical knowledge.

The Fairfaxes were not unusual in keeping recipe books in the family. A trawl through hundreds of surviving manuscript recipe books reveals that many early modern families also adopted the practice of passing down useful household knowledge from one generation to another. Recipe books, in some cases, were considered worthy enough to be mentioned in wills and bequests alongside other household objects of value.^18^ For example, on 7 March 1704, just under one year before her death, 84-year old Lady Johanna St John wrote out her will in her own hand.^19^ The will contains a number of personal bequests – she left her bible to her eldest son, various pieces of furniture, paintings and silver to her daughters and granddaughters and small gifts of cash and linens to old, loyal servants. Alongside these gifts was the mention of two recipe books. The first was a ‘book of receipts of cookery and preserves’ left ‘according to [her] promise’ to her granddaughter Soame and the second, gifted to her daughter Cholmondley, was her ‘great receipt book (St. John, 1704, fol. 300v).’ The specific listing of these two notebooks underlines the value (monetary and otherwise) placed upon recipe books both as material objects and as collections of knowledge.

**Fig. 1:**
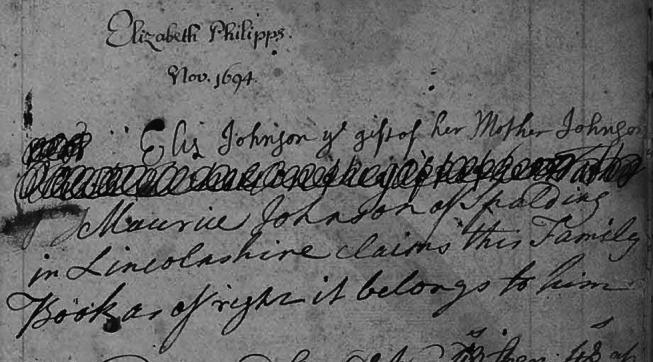
Wellcome Western MS 3082, fol. 27r.

In some cases, such as the Johnson family from Spalding, Lincolnshire, the second generation felt that it was their right to inherit the ‘family book.’ Maurice Johnson, an antiquarian and barrister, was particularly keen to assert his claim to his stepmother's book of recipes. The volume now has three ownership notes (Figure 1). The first ‘Elizabeth Phillipps Nov. 1694’ was written before Elizabeth Oldfield Philips married into the Johnson family. The second ‘Eliz Johnson ye gift of her Mother Johnson’ most likely refers to Elizabeth Johnson's daughter-in-law and the final note was written by her son Maurice Johnson the younger. It reads ‘Maurice Johnson of Spalding in Lincolnshire claims this family book as of right it belongs to him.’^20^ Johnson's enthusiasm for household practical knowledge may be related to his other intellectual interests, as he was a founding member of the Gentlemen Society of Spalding, Lincolnshire. The society is one of the oldest such organizations in England and in its heyday boasted both a museum and a physic garden (Haycock, 2004). After claiming it as his own, Johnson and his immediate family certainly made good use of the ‘family book.’ It became the work of a large number of compilers who reviewed and annotated upon particular recipes and new information was still being added in the mid nineteenth century.^21^

Another mother, Lady Frances Catchmay, took no chances in assuring that all her children would inherit the practical household knowledge she gathered over the years. A copy of her collection bears this inscription on the first folio:

this booke with the others of medicins, preserves and cookerye, my lady Catchmay lefte with me to be delivered to her sonne Sir William Catchmay, earnestly desiringe and charginge him to lett every one of his brothers and sisters to have true copyes of the sayd bookes, or such parte thereof as any of them doth desire. In witness that this was her request, I have thereunto sett my hand at the delivery of the sayd bookes. Ed. Bett (Catchmay, verso of first preliminary leaf.)

Thus, the gift of the ‘family books’ to William Catchmay came with the responsibility to disseminate the knowledge contained within. Notably, both medical and culinary knowledge were included in this bequest, reminding modern readers of the close association between food and medicine in the early modern period.^22^ Catchmay was not alone in using the bequest of her recipe collection to continue influencing and expressing hopes for her children. Another demonstration of this practice is the intriguing inscription on the title page of Folger manuscript v.a. 430 which reads:

Mrs. Ann Granvills Book which I hope shee will make a better use of then her mother Mary Granville^23^

Underneath this inscription is written ‘Now Anne Dewes/ Bradley 8^th^ Sept 1740.’ Mary Granville's note tantalizingly hints at her feelings of guilt over her own lack of interest, or perhaps skills, in housekeeping. Yet at the same time, it betrays this mother's aspirations for and sense of responsibility to guide her daughter in the assigned role of housewife.

Early modern fathers also hoped that their daughters would make good use of the household knowledge passed on to them. On 2 October 1610, the widower Valentine Bourne inscribed his name in what would later become a miscellany of medical texts and family and local history (Bourne, fol. 1r). Over the next 20 years or so, Bourne added not only medical, veterinary and culinary recipes but also other relevant medical information including conversion charts of weights and measures, a glossary of difficult medical terms, a treatise on urinoscopy, an extract from Galen's *De alimentorum facultatibus* and a section on preserving health.^24^ Besides the medical information, Bourne recorded both family and local history, recording the dates of birth, death and marriage in his family from his own year of birth in 1566 to the death of his father-in-law in 1626. He also copied out lists of the Mayors and Sheriffs of Norwich between 1403 and 1648 and of all the high Sheriffs from the reign of Elizabeth to 1660.^25^ Twenty-six years after Bourne began this notebook, he bequeathed it to his lovinge daughter Elizabeth.^26^ Within the recipe portion of the book, blank spaces were left at the end of each section, suggesting that Bourne harbored hopes that his daughter would carry on his interests in gathering medical information and continue to expand the collection with new recipes.

Another father, Sir Peter Temple of Stantonbury in Buckinghamshire, also shared Valentine Bourne's view that it was necessary to compile a general medical guide for his daughter (Temple a and b). Temple's notebook for his daughter Elinor included both a collection of medical recipes and a set of instructions on how to approach and use the material provided Temple a, fols. 10 r-v). These instructions consisted of information on the origin of the recipes and the trustworthiness of the authors, a brief general method for the preparation of medicines and short explanations about the way in which the book was arranged. Not only was Elinor continually reminded of her father's presence by his handwriting, Temple also extended his influence throughout the text with short notes of advice and endorsements of particular remedies all signed with his initials ‘P. T.’.

The creation of a recipe book for a particular family member could also work posthumously, *in memoriam* rather than for actual use. Lady Mary Fane organized her mother Grace Mildmay's ‘divers books and more than 2,000 loose papers’ and had them copied as one volume. Mary referred to these papers as ‘the treasure…of my worthy mother's mind’ and viewed them in the same light as the ‘other worldly inheritances and goods’ which were passed to her upon Grace's death. Mary Fane undertook the collection of papers which were ‘scatteringly and confusedly left’ and the delivery of them as one volume ‘digested into four books’ because she was ‘desirous and careful to commend [them]…to her [mother's] posterity's view and imitation.’ 27 For Mary Fane, the inheritance of knowledge came with a responsibility to sort, organize and preserve.

Mary Fane, of course, had inherited not just medical papers but also autobiographical reflections and spiritual meditations. Grace Mildmay had constructed an archive of family records for her daughter and grandchildren. Other compilers also joined records of family information with recipes. One such notebook belonged to the Napier family of Holywell, Oxfordshire. Like both Fairfax family notebooks, this leather-bound notebook has the initials of the original owner stamped on the front cover. Here, the initials stand for Elizabeth Powell who, as the first owner of the book, brought the book to Oxfordshire when she married William Napier. After her death, the book passed through the hands of two of her sons, Christopher and Edmund, into the possession of her grandson George before finally ending up in the house of her great-granddaughter Margaret Napier Nevill.^28^ Unlike the Fairfaxes, each generation of the Powell/Napier/Nevill family left their imprints on the notebooks not only in the form of recipes but also in the form of other information. The notebook now contains a miscellany of family and financial information including a list of births and deaths in the Napier family and copies of letters and accounts relating to the estate of George and Margaret Napier.

The pairing of household information with family records can also be evidenced in other recipe books. Anne Glyd's notebook also combined family records with medical and culinary recipes (Glyd).^29^ Titling the section ‘A memorial of our childrens births,’ Anne carefully noted the birth dates of her eight children and five of her grandchildren. In each entry recording the death of a child, Anne, keeping meticulous track of time, recorded their age to the week. On the passing of her adult children, John and Elizabeth, Anne was careful to acknowledge God's grace in aiding both to be good, gracious and ‘useful…to man.’ On the birth of each of her grandchildren, Anne expressed her gratitude to God and also put forward her hopes for the child's future. Anne's book now lies within the family papers of her son-in-law Sir William Brockman, suggesting that it was given to her youngest daughter, Anne, to keep within the family. Anne Glyd Brockman inherited not only her mother's medical knowledge and a copy of her family records but also expressions of her mother's hopes and dreams for her children and grandchildren.

The miscellaneous information of the Bourne, Napier and Glyd-Brockman family notebooks recalls the earlier Italian genre of *libri di famiglia* or *ricordanze* (Cicchetti and Mordenti, 1985), the familial notebooks in which the patriarch of the family recorded dates and city-wide events affecting the family and copies of financial and legal documents. Functioning as family archives, these notebooks were closely guarded objects within particular families and were passed down the patrilineal line. The presence of similar information in these English notebooks suggests that recipe books might have also served a dual function within seventeenth-century English households. That is they acted both as a repository of practical, useful knowledge and as a family archive. While the transmission of the earlier *libri di famiglia* might have been strictly patrilineal, in England, by the sixteenth and seventeenth centuries, women were often the keepers of genealogical information (Woolf, 2003, pp. 116–117). Extant manuscript miscellanies connected with female compilers suggest that it was a fairly common practice to write family records alongside other information. In addition to medical and culinary recipes, family records can also be found mingled among household accounts, rent receipts, copies of sermons and reading notes (Sherman, 2008, p. 58). Female readers also annotated and recorded family information within books such as Bibles, the Book of Common Prayer and books devoted to childbirth (Sherman, 2008, ch. 3). While they may not contain formal family trees, the two Fairfax family books can also be seen as part medical notebook and part family archive, for they record not only the medical interests of the Fairfax clan but also their medical histories. At the same time, a skeletal family tree can also be reconstructed by tracing the owners and the many contributors of the two books. For each generation of the Fairfax family, the volume must also have represented a piece of family history.

Household recipe books highlight the complex gendering of inheriting and bequeathing household knowledge. Conventionally, historians have emphasized the bequest of recipe collections down matrilineal lines. For example, Jennifer Stine and Sara Pennell have pointed out that many of these collections were given as wedding presents or were brought by women from the parental home to the new household (Stine, 1996, p. 111; Pennell, 2004, pp. 240–241). While that is no doubt true in many cases, as evidenced by Mary Cholmeley and Rhoda Hussey's recipe books, it was by no means the only way in which collections changed hands. As the manuscripts of the Napier, Johnson, Catchmay, Bourne and Temple families demonstrate, men were clearly involved in the process of creating medical knowledge for the next generation and in receiving knowledge from their parents. Even in the cases where the book seems to have passed through matrilineal lines (such as the two Fairfax family manuscripts), the book's life journey may not have been that simple. Both Fairfax family manuscripts actually travelled through the hands of a son before returning to female ownership. Mary and Henry Fairfax's book was first given to their eldest son Henry Fairfax before passing to their granddaughter Anne and Rhoda Hussey Fairfax's book passed through her grandson Thomas Cartwright's hands before following his daughter Ursula Cartwright to the Skipwith family at Newbold Hall. The practice of bequeathing recipe books and the important role that inheriting knowledge played in the transfer of these books complicates the association of such information with gendered areas of knowledge. Consequently, tracing the *fortuna* of individual manuscripts encourages us to challenge traditional boundaries surrounding spheres of knowledge. While historians have often connected the collection and use of household knowledge with the domestic sphere, housewifery and the female domain, it appears that such knowledge and practices cannot be so neatly categorized.

## 3. Starting Out

The practice of passing down household recipe books from generation to generation, thus, brought with it a multi-stage and open-ended construction process. As the owners adopted the role of a compiler and began to adapt the book to suit their needs, they left traces of their collecting activities behind in the form of their own distinctive handwriting. With the relevant information to identify the handwriting of different compilers, these texts can be peeled back layer by layer. Thanks to the extensive papers left by the Fairfax family and to George Weddell's work on identifying hands in Henry and Mary Fairfax's recipe book, the *Arcana Fairfaxiana* can be deconstructed in just such a way. The collection was compiled in three distinct phases: (1) the original book brought to the Fairfax family by Mary Cholmeley, (2) Mary, Henry and their immediate family's contributions and (3) later eighteenth-century additions.

The two recipe books which accompanied Mary and Rhoda Fairfax to their new homes were not blank books but rather contained a small number of recipes with copious blank or ‘waste’ pages waiting to be filled with practical know-how.^30^ Mary Cholmeley's book begins with two sections of recipes written in a practiced cursive hand. Each recipe within these two sections was set out with clear, defined headings and separated by decorative lines. The first section, at the front of the volume, contains a series of medical recipes addressing a range of ailments (Fairfax Family, 1890, pp. 9–58). The second section consists of a series of recipes taken either from an apothecary's formulary or a physician's notes.^31^ Within these two sections, if a recipe spanned across more than one page, catchwords were used, suggesting that the text was first copied and then bound. This same hand also penned a note on ‘Miss Barbara's’ lessons on the virginal and a list of late sixteenth-century musicians on the back fly-leaf. Like Mary's book, Rhoda's book also starts with a neatly copied set of recipes. Titled ‘Mris Anne Brumwich her Booke of Receipts or Medicines ffor severall sores and other Infermities’ this set of recipes was organized alphabetically by ailment, beginning with recipes for the ague and continuing with remedies for consumption, cough, etc. At the end of each alphabetical section and within the index, spaces and blank pages were left for later additions. An index at the end of the volume completes the copy. Of course, these neatly written sections did not represent the entirety of the Fairfax family's recipe knowledge. In both the books, Fairfax family members filled the empty pages with their own gathered recipes. These are slotted in within the existing organizational schemes and the index or table of contents were updated accordingly. The two books as they exist now both have almost alternating sections of the neat scribal hands of initial sections and the more freeform handwriting of Fairfax family members.

**Fig. 2:**
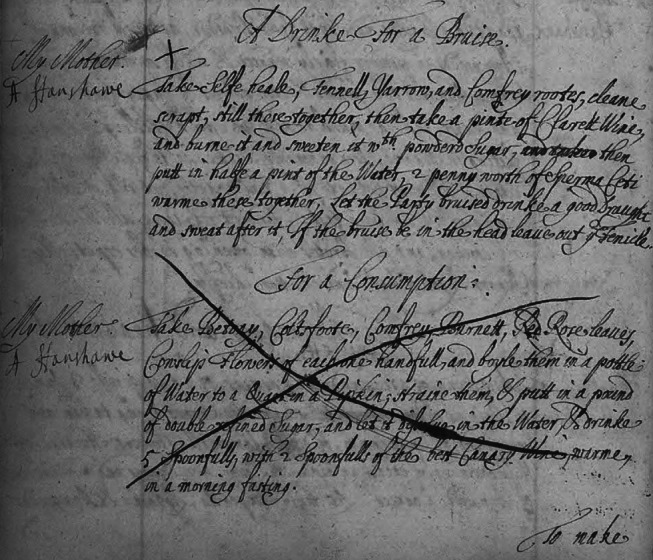
Wellcome Western manuscript 7113, fol. 4r.

At this junction, it is useful to think of the neatly written sections of these books as ‘starter’ collections – an initial trove of household knowledge to give the Fairfax's a start in their recipe gathering activities. Not surprisingly, these ‘starter’ portions are a common occurrence in early modern recipe notebooks. The origin of these ‘starter’ portions varied from case to case. As we saw above in the cases of the Temple and Bourne family books, sometimes a family member might take the time to copy out the ‘starter’ portion. In other cases, compilers might themselves take the initiative to create these books either by doing the copying themselves, or as in the case of Lady Anne Fanshawe, by commissioning a scribe, Joseph Averie, to make a copy of her mother Margaret Harrison's collection.^32^ After the copy was completed, Anne herself read through the manuscript, annotating as she went along. As illustrated in Figure 2, Fanshawe used a range of common reading annotations such as ‘X’ to mark out particular recipes. She also crossed out recipes that failed to meet her expectations and signed her name against recipes that she had tried and tested.

**Fig. 3:**
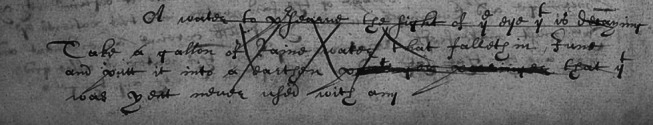
Wellcome Western manuscript 160, fol. 3v.

Fanshawe's collection reminds us that ‘starter’ collections could be inherited. However, despite the value placed upon inherited knowledge, it was not deemed trustworthy merely by its lineage but rather still awaited evaluation and testing. A similar practice can be seen in the *Arcana Fairfaxiana* which bears traces of Henry's and Mary's interaction with their inherited ‘starter’ collection. This can be seen throughout the ‘starter’ portion of the manuscript where they marked up recipes such as the ‘X’ written next to a recipe ‘For Bleeding at ye nose.’ Henry, in his distinctive handwriting, also commented upon recipes. For example, he wrote ‘& is good for bleeding’ under the title of a medicine for a laske (looseness of the bowels) (Fairfax Family, 1890, p. 11). Likewise, Rhoda Fairfax and her descendents questioned Anne Brumwich's knowledge. As shown in Figure 3, they too decided to cross out recipes that failed to meet their needs.

For compilers of early modern household books, the adoption of ‘starter’ portions was much like getting a ‘kick-start’ in creating their own collections of medical knowledge. The presence of these ‘starter’ collections indicates that both the producer/donor and the receiver/future compiler saw a need for a ready-made set of general recipes. Yet this need for general information was paired with a desire to personalize and adapt these collections to one's own requirements – hence the blank spaces left in each collection and the subsequent evaluation of the information contained therein. Household recipe collections were by nature ever-expanding books of knowledge which changed according to the needs of the current owners.

## 4. A Household Affair

For many early modern men and women, inheriting or obtaining a notebook of medical and culinary recipes was just the first step in creating their own treasury of useful information. Once the *Arcana Fairfaxiana* arrived at Mary Cholmeley Fairfax's new home, it entered its second phase of production, the stage at which Henry and Mary took the ‘starter’ collection, added to it and customized the existing information to make it their own. If adding to a ‘starter’ collection can be seen as collaborative compilation across time, Fairfax family members also worked collectively on the book within the same time frame. During this second phase of compilation, most of the recipes were accumulated and entered one by one. Some were slotted into blank spaces within the ‘starter’ collection, often amongst information dealing with a similar ailment, but the majority of these single recipes were written in the blank pages of the notebook. Henry's tiny cursive hand is dominant in this period and can be seen throughout the different sections of the book. He not only asserts his ownership of the volume by inscribing his name but also wrote in a Latin epigram with translation, compiled the front and back indices, added in information on weights and measures and inserted a number of recipes within the ‘starter’ section of the text (Fairfax Family, 1890, pp. 1, 2, 5–7, 194–198, 8 and 38).

While Mary Cholmeley Fairfax may not have been as enthusiastic a compiler as her husband, she also wrote in a number of recipes interleaved within those copied by Henry. One of her entries, a medicine for wind attributed to Queen Elizabeth, has corrections and additions in Henry's hand (Fairfax Family, 1890, p. 63). Mary's version instructed the reader to ‘ponde [the ingredients] together;’ Henry added above this ‘& searce them.’ He also felt the need to add ‘it expels winde’ to the list of virtues given by Mary. Henry also annotated other recipes written by Mary including a recipe to make ‘the green oyntment.’ Here, Mary wrote that one should take ‘red sage and rewe of ech a quart,’ and Henry added above ‘a pound or [a quart].’ The quantity of two other herbs used in the recipe, bay leaves and wormwood, was given in pounds and thus Henry may have added the extra measurement to ensure consistency (Fairfax Family, 1890, p. 65). The interaction between Henry and Mary not only gives us a sense of their personalities but also emphasizes the collaborative nature of creating a household book of recipes.

The *Arcana Fairfaxiana* saw not only the pens of Henry and Mary but also those of other members of their immediate family. Brian Fairfax, Henry and Mary's younger son, wrote a section close to the end of the book. His entries included a drink for the plague, a copy of the instructions to make Dr Chamber's water and a series of miscellaneous recipes (Fairfax Family, 1890, pp. 151–153). Mary's brother Henry Cholmeley contributed a large number of recipes some written in his own hand and others neatly entered by Henry Fairfax.^33^ Henry Fairfax's own brother Ferdinando also wrote a recipe ‘For a could’ in the book. However, he neglected to sign his name to the information and the attribution was later provided by the diligent Henry Fairfax (Fairfax Family, 1890, p. 146). Perhaps returning the favor, Henry himself contributed to Ferdinando and Rhoda's book where he wrote, in his distinctive hand, recipes to make ‘walnut water’ and a cordial water (Fairfax, p. 111). The recipes that the two brothers wrote in each other's books do not overlap with information within their own household books, enticing readers to wonder about the exact circumstances that prompted these entries. Whatever the reasons behind Henry and Ferdinando writing in each other's recipe collections, their entries emphasize the importance of familial collaboration in recipe compilation and point to the openness of household books to contributions from members outside the nuclear family unit.

In fact, Henry and Mary Fairfax were not shy in utilising a number of different knowledge networks to extend their collection. On the family front, Henry and Mary gathered recipes from various members of the extended Fairfax and Cholmeley clans including a cluster of recipes taken from Lady Sheffield, the maternal grandmother of their niece Dorothy Hutton.^34^ The Fairfax's also collected recipes from men they addressed as ‘Dr’ including physicians such as Drs Butler, Chambers and Stevens, whose recipes were circulating widely in the period. Finally, they perused contemporary medical books for information and copied several recipes from the Italian ‘professor of secrets’ Leonardo Fioravanti.^35^ Consequently, while the inheriting and bequeathing of recipe books was confined, for the most part, within nuclear family structures, the expanding of a recipe collection involved a myriad of different actors and compilers and often called upon members of the large household-family circle for their knowledge and connections. This suggests that there was a substantive difference in the way early modern men and women viewed recipes as opposed to recipe books. The leather or vellum bound books of recipes were seen as heirlooms – material objects to be bequeathed to the next generation within the narrow confines of a lineage family. Single recipes, on the other hand, as tidbits of knowledge lacking the materiality of an actual book, circulated in a much wider community.

## 5. Gendering Household Knowledge

From the two Fairfax family books to Mary Fane's organization of her mother Grace Mildmay's papers to Peter Temple and Valentine Bourne's gifts to their daughters, the recipe books discussed in this chapter bring to mind not housewifery guides authored by individual women but household books filled to the brim with the collective knowledge of a family. Husbands and wives, fathers and mothers, brothers and sisters all contributed to, wrote in and owned these books. Sons and daughters inherited the books and the practical knowledge contained within. As new owners sought to individualize and customize the books to their own and their family's needs, they collected and added new recipes and tested and adapted old ones. The result was a book which lay at the heart of the household, a central place to record practical medical and culinary knowledge. When seen from this angle, it is not surprising that a number of compilers combined family history with recipes, for in some ways the recipe book was a kind of family archive. For modern readers unfamiliar with the many different hands in each volume, the archaeological layers may be identifiable but undecipherable, but for a family member in the age of letter writing, the many different hands would have clearly signaled individual sources and perhaps even particular occasions. For early modern men and women, inheriting the ‘family book’ meant receiving tried and tested practical knowledge as well as a physical record of their family lineage. Practical household know-how was thus closely intertwined with family history. After all, the creation of many of these books, such as that of Henry and Mary Fairfax, paralleled the building and growth of a family. The myriad of open-ended ‘starter collections’ inherited by early modern men and women embodied hopes and dreams of accumulating not only medical knowledge but also progeny. For early modern men and women, reading recipes might have been one way of recollecting and imagining the past.

The collaborative authorship of so many of these household recipe books encourages modern readers to question past notions of authorship associated with the genre. The complex steps through which these books were constructed show that assigning a single author to these types of texts is misleading and oversimplifies the knowledge production process at work. It is perhaps useful to follow Maurice Johnson the younger and refer to the texts as ‘family books’ to highlight the collective efforts of household members over several generations. Cross-generational collaborative compilation also has implications for knowledge ownership. The entire family was responsible for and felt ownership of their book of practical knowledge. In return for benefitting from the collecting efforts of the previous generation, users had to pay a contribution in the form of new knowledge. Thus, these ‘family books’ functioned as more than just treasure troves of knowledge; they worked to bind family units together. While the actual medicaments described in these books may have been similar in form and content to those available commercially, the central role played by family and household in the creation of these books meant that practical knowledge created within the domestic space took on an additional social dimension.

The uncovering of family-wide involvement in gathering and generating practical knowledge also encourages a rethinking of the gendering of household medical and scientific activities. Historians of science have highlighted that as a site of knowledge production, the early modern household was one which particularly fostered collaboration between different household and family members.^36^ These collaborations often involved men and women occupying various positions within the household structure working alongside each other. In the ‘family books,’ we not only see husbands and wives working together as in Londa Schiebinger's case of Gottlieb and Maria Kirch-Winkelmann but we also see extended family members, household servants and dependents acting as knowledge contributors and aiding medicine production in a myriad of ways from setting up the distillation equipment to stoking the long burning fires to cultivating the necessary herbs.^37^ By acknowledging that entire household-families played a part in creating these books, we open pre-modern household medicine and science to wider participation.

This essay has chosen to foreground the household collective in informal knowledge making; however, while the picture I paint may be that of happy families, that is not to say that gender was not a consideration within this process or that there were not potention tensions or gender conflicts. Boundaries surrounding both who could be involved in these practices and their assigned roles were fluid and flexible. Like many things within a family, these were up for constant negotiation and shifted according to personal interests of family members and the multiple overlapping positions they occupied within the household. The case of recipe ‘family books’ is marked out by the fact that health concerns affected the wellbeing of an entire household and thus it was in the interests of the household collective to utilize the knowledge base, the individual skills and the information channels of all members, in many cases regardless of their gender. However, contemporary views towards women's involvement in household medical activities were not straightforward and gender remains a category which men and women used to assess knowledge claims. A clear articulation of these tensions is provided by Peter Temple. As discussed earlier, Temple took the time to create a recipe book for his daughter Eleanor thereby confirming her role as a healthcare provider and as a ‘knower’ of medical knoweldge. Yet, in the same volume, he also openly questions the trustworthiness of information collected from female sources. He writes in the explanatory notes accompanying the notebook: ‘[a]lthough I often have these receits from women, yet men of Judgment make them; (as the La: Forster receits come most from Sr Theador Maynerne.) & such are usually harmelesse, & certaine (Temple a, fol. 10r).’ While Temple's gift to Eleanor signals his approval and encouragement towards women engaging in these sort sof activities and his willingness to collect recipes from women indicates that he sees them as a potential sources of new knowledge, ultimately, he remains wary of their claims as knowers.

The *fortuna* of these two family books from their Yorkshire homes to their eventual rediscovery by two nineteenth-century chemists has allowed us to uncover a narrative of informal science. The main actors involved in the production and transmission of these books all conducted their practices of natural inquiry outside the academy. While our early modern householders were joined in their endeavors by a range of other ‘experimenters’ working in various non-academic spaces, by the time of our nineteenth-century chemists, the spaces of doing science were much more delineated. Working in the commercial sphere, both our chemist entrepreneurs sought to gain entry into the academy. Weddell advertised his books in academic journals in the fields of medicine and history and Wellcome used his wealth to amass a collection of artifacts in part to try and position himself within the scholarly community.^38^ Interestingly, by this time, despite their training as chemists, Weddell and Wellcome did not lay claim to be experts of contemporary medicine or science but rather as knowers of histories of medicine. Through the endeavors of Wellcome and Weddell, both the Fairfax recipe books ventured out of the family-orientated sites of early modern kitchens and homes into new public sites of knowledge production: printed books and journals, libraries and museums.

Centered on the two Fairfax family books, this essay has argued that recipe collections, as records of home-based natural inquiry, provide a glimpse into knowledge transfer and production within early modern English households. As texts created collaboratively by family collectives across gender, geographic and temporal boundaries, they extend our understanding of the multiple roles assigned to men and women within early modern households and how these roles provided opportunities for family members to participate together in knowledge-making activities. Finally, their survival, one in a country house in rural Warwickshire and another in the busy shop of an urban chemist, tells the tale of how informal science has functioned in parallel to the goings-on in the academy.

## NOTES

1 The firm in question is Mawson, Swan and Weddell, a long established chemist and druggist firm in Newcastle which counted John Mawson and Sir John Wilson Swan as past owners. Both Swan and Weddell had wide-ranging scientific interests. In the early 1880s, Swan left the pharmacy trade and began developing and selling early electric lamps for the Edison and Swan company. Weddell is now best known as the inventor of the Cerebos/Saxo brand table salt. See Brown (2004) and Corley (2004).

2 Interestingly, the premises were previously occupied by another chemist: Gilpin & Company. George Weddell describes his discovery of the Fairfax manuscript in his introduction, (Fairfax Family, 1890, p. ix).

3 For Henry Wellcome's collecting strategies, see Larson (2009, chs. 5 and 6). For a biography of Ferdinando Fairfax, see Hopper (2004a).

4 The majority of the manuscript recipe books cited in this essay were compiled seventeenth century. In order to keep the in-text citations brief, I will cite only the names of the collective authors. Full references are available in the bibliography.

5 Weddell writes in his introduction that the Fairfax family book ‘enable[s] us to more readily call up the family life of those interesting days in or about 1600’ and he also encourages readers to try out the housewifery tips presented in the book: *Arcana Fairfaxiana*, pp. xlii–xlvi (xlv).

6 C. J. S. Thompson first started to work for Wellcome on the ‘Animal Substances’ book and his first book purchase for Wellcome was another seventeenth-century English household recipe book (Ayscough). The purchase of Rhoda Fairfax's book in 1907 occurred during the period when Thompson was actively working on the ‘Historical Exhibition’ project: (Larson, 2009, chs. 3 and 5).

7 See Moran (1991); Smith and Findlen (2002); Smith (2004); Ogilvie (2006); Barrera-Osorio (2006); Eamon (2006) and Roberts, Schaffer and Dear (2007).

8 For households as spaces of natural inquiry see Cooper (2006), Hunter and Hutton (1997), Harkness (1997), Pennell (2004), Rankin (2007) and Leong (2008).

9 For an overview, see Wear (2000). Lucinda Beier's study of the Josselin family provides a good illustrative case study: Beier (1985). See also Leong (2008).

10 The literature on pre-modern recipe collections is vast. Select works include Totelin (2009), McVaugh (1971, 1976) and Green (2008) (especially Chapter 4 and conclusion). A key work on early modern recipes and ‘secrets’ genre is Eamon (1994). For a recent overview of the genre within a Europe-wide setting, see essays in Leong and Rankin (2011). For a description of Victorian pharmacists and household recipes, see Marland (2006).

11 In recent years, there has been a flurry of research on English recipe books and on English manuscript recipe books in particular. Pioneering works include (Beier (1987, ch. 8); Nagy (1988, ch. 5); Stine (1996); Hunter (1997a,1997b). More recent works include: Wear (2000, ch. 2), Pennell (2004), Leong and Pennell (2007) and Leong (2008). Studies which focus upon analyzing recipe collections as a genre of women's writing include Knoppers (2007); Ezell (2007); Field (2007); Spiller (2010).

12 Guthrie (1913), Spurling (1986) and Schoonover (1998). Lady Sedley's recipe collection is now in the library of the London Royal College of Physicians (RCP MS 534). Other manuscript collections which have been reproduced in modern editions include Blencowe (1925); Price (1974); Chafin (1979); Hess (1981); Evelyn (1997).

13 Despite the fact that most of the texts analyzed by Theophano and Pennell contain definite medical elements, both authors elected to present these texts as ‘cookbooks’: Theophano (2002, p. 51) and Pennell (2004).

14 The notion of collaborative authorship is received considerable attention in recent years. For a helpful summary of recent work, see Smith (2012, pp. 4–5) For a focused discussion on collaborative authorship and recipe books, see also Field (2007).

15 The literature on early modern English family structures is abundant. A key recent work is Tadmor (2001). Tadmor argues that ‘family’ in the minds of early modern men and women could mean ‘a household, including its diverse dependants, such as servants, apprentices and co-resident relatives.’ She introduces the concept of the household-family to refer to this structure arguing that ‘the boundaries of these household-families are not those of blood and marriage, they are the boundaries of authority and of household management,’ pp. 19 and 24.

16 Fairfax, pp. 171 and 180. The loose recipe was inserted between 170 and 171. I have not been able to locate biographical information on Dr Catlin.

17 Cocken is in county Durham.

18 It was somewhat unusual for early modern English women to write wills as under the doctrine of coverture married woman's legal identity was absorbed with her husband's. Johanna St. John's will, written in her own hand, clearly states her husband Sir Walter St John's consent at the start of the document. Due to the scarcity of women's wills in early modern England, it is difficult to get a sense of whether it was common for women to bequeath recipe books. For more information on women, property and wills, see Erickson (1993) and Prior (1990).

19 Johanna St. John of Bletso was the daughter of Oliver St. John (1598–1673), Lord Chief Justice to Oliver Cromwell. She married a kinsman Sir Walter St. John of Lydiard Tregoze, M.P. for Wiltshire (1621–1708) and was grandmother to Henry St. John, first Viscount Bolingbroke (1678–1751). See Palmer (2004) and Dickinson (2004). See also St. John (1704) and Smallwood (1969). The ‘great receipt book’ is now in the Wellcome Library, London: St. John.

20 Johnson, fol. 27r. The Johnson family book was most probably begun by Elizabeth Oldfield Philips Johnson in 1694 during her first marriage to Andrew Philips of Leominister. Like the brides mentioned above, Elizabeth Philips brought the book with her to Spalding following her marriage to Maurice Johnson. For a detailed family tree, see Green (1877).

21 The Johnson family book is well annotated. The first section of the manuscript contains miscellaneous material including newspaper cuttings dating to the first quarter of the nineteenth-century: see, for example, Johnson family, fols. 3r (a recipe to destroy moths dating to 1827), 6r (information on growing strawberries dated 1828) and 27v (notes from various newspapers and a clipping of a recipe for ‘Otto of Roses’).

22 See, for example, O'Hara May (1971) and Pelling (1998).

23 Granville, pastedown on front cover. The notebook was compiled between 1640 and 1750. Mary Granville was the daughter of Sir Martin Wescombe, who was the English Consul in Cadiz, Spain from 1663 to 1688. Anne Granville D'Ewes married John D'Ewes of Wellesbourne, Warwickshire and resided in Bradley, Worcester: Field (2007, p. 55).

24 Bourne, fols. 62r-131v. (medical recipes), 33r-61r (culinary recipes), 169v-73v (veterinary recipes), 4r-v (weights and measurements), 6r-8v (glossary), 200v-194v (treatise of urine), 205v-1v (extract of Galen's *De alimentorum facultatibus*)and 209v-7v (section for preserving health). The book was turned upside down and information was entered from the back of the volume, hence some folio numbers are reversed.

25 Bourne, fols. 9r-10r (family records), 187v-73r (list of Mayors and Sheriffs) and 193v-88r (list of high sheriffs). Bourne, here, does not appear to be attempting to construct a local history of Norwich or any such similar ambitious project. The recording of local historical and genealogical information was commonplace practice. For a detailed discussion of early modern historical culture, see Woolf (2003).

26 Bourne, fol. 9r. Elizabeth was born in 1599 and thus was in her mid-thirties when the notebook was finally passed to her.

27 Mildmay, fol. 3r. For additional information on Grace Mildmay, see Pollock (1993).

28 Napier. The volume has inscribed on the first folio: ‘sum liber edmundi ex dono christoferi napperi.’ The Nappers or Napiers were a prominent Oxfordshire family from Holywell. Both Edmund (1579–1654) and Christopher were sons of William Napier (bur. 1621–1622) and Elizabeth Powell Napier (bur. 1584). Edmund Napier's son George (bap. 1619–1671) married Margaret Arden of Kertlington. George and Margaret's daughter Margaret married Henry Nevill of Holt in Leicestershire: Squibb (1993).

29 The book was most probably brought into the Brockman family when Anne's youngest daughter, also Anne, married William Brockman. The brief family history section can be found on fols. 82r-84r. There are a number of recipe books within the Brockman Papers in the British Library. In addition to Anne Glyd's book, there is also a loose collection of recipes associated with Anne Glyd and Anne Brockman (Additional MS 45198); a notebook of recipes and French exercises with the ownership note ‘Eliz Brockman 8 May 1674’ (Additional MS 45199, fol. 1r) and a recipe collection associated with Anne Bunce Brockman (d. 1660) dated to 1638 (Additional MS 45197). For additional information on Anne Bunce Brockman, see Aughterson (2004).

30 The practice of leaving blank leaves within notebooks is a fairly common one in early modern England. These leaves are often referred to as ‘waste leaves.’ See Vine (2011).

31 The recipes within this section differ in format from those in the first section as each entry lists only the recipe title and the Latin name and required quantity of the ingredients used: Fairfax Family (1890, pp. 75–96).

32 Fanshawe, fol. 3r.

33 Henry Cholmeley's recipes covered a wide-range of ailments and are jotted throughout the book. The main section, written in Henry Fairfax's hand, can be found on Fairfax Family (1890, pp. 138–141).

34 Dorothy Hutton was the daughter of Henry's brother Ferdinando (1584–1648) and his first wife Mary Sheffield (d. 1619), the daughter of Edmund Sheffield, the first earl of Mulgrave and his wife Ursula. The Lady Sheffield mentioned in the book most probably referred to Ferdinando's mother-in-law: Hopper (2004a). For a list of contemporary authors and their possible realtionship to Henry and Mary Fairfax, see Fairfax Family (1890, pp. xxviii–xxix).

35 Fairfax Family (1890, p. 62). For additional information on Fioravanti, see Eamon (2010).

36 See, for example, Cooper (2006), Schiebinger (1989) and Fissell (2008).

37 Schiebinger (1989, ch. 3). See also Shapin (1989).

38 Weddell advertized the volume in the current issues of *The Library, The Lancet* and *The British Medical Journal*. The book was favorably reviewed in *Notes and Queries* (7th series, 1891, 100 and 181–182) and *The British Medical Journal* (June, 1891, 1235–1236). For Wellcome, see Larson (2009, ch. 5).
